# Genetic diversity and evolutionary history of Korean isolates of severe fever with thrombocytopenia syndrome virus from 2013–2016

**DOI:** 10.1007/s00705-020-04733-0

**Published:** 2020-07-22

**Authors:** Mi-ran Yun, Jungsang Ryou, Wooyoung Choi, Joo-Yeon Lee, Sun-Whan Park, Dae-Won Kim

**Affiliations:** 1 Pathogen Resource TF, Center for Infectious Diseases Research, Korea National Institute of Health, Korea Centers for Disease Control and Prevention, 200 Osongsaengmyeong2-ro, Heungdeok-gu, Cheogju-si, Chungbuk 28160 Republic of Korea; 2 Division of Emerging Infectious Disease and Vector Research, Center for Infectious Diseases Research, Korea National Institute of Health, Korea Centers for Disease Control and Prevention, 187 Osongsaengmyeong2-ro, Heungdeok-gu, Cheogju-si, Chungbuk 28160 Republic of Korea; 3 Division of Viral Diseases, Center for Laboratory control of Infectious Disease, Korea Centers for Disease Control and Prevention, 187 Osongsaengmyeong2-ro, Heungdeok-gu, Cheogju-si, Chungbuk 28160 Republic of Korea; 4 Jeju National Quarantine Station, Korea Centers for Disease Control and Prevention, 356 Central Goverment office-Jeju, 59 Cheongsa-ro, Jeju-si, 63219 Republic of Korea

## Abstract

Severe fever with thrombocytopenia syndrome (SFTS) is caused by SFTS virus (SFTSV). Although SFTS originated in China, it is an emerging infectious disease with prevalence confirmed in Japan, Korea, and Vietnam. The full-length genomes of 51 Korean SFTSV isolates from 2013 to 2016 were sequenced, and the sequences were deposited into a public database (GenBank) and analyzed to elucidate the phylogeny and evolution of the virus. Although most of the Korean SFTSV isolates were closely related to previously reported Japanese isolates, some were closely related to previously reported Chinese isolates. We identified one Korean strain that appears to have resulted from multiple inter-lineage reassortments. Several nucleotide and amino acid variations specific to the Korean isolates were identified. Future studies should focus on how these variations affect virus pathogenicity and evolution.

Severe fever with thrombocytopenia syndrome (SFTS) is caused by SFTS virus (SFTSV), a member of the order *Bunyavirales*, family *Phenuiviridae*, genus *Bandavirus* (https://talk.ictvonline.org/taxonomy). SFTS is a newly emerging infectious disease with its major clinical symptoms and laboratory findings including fever, thrombocytopenia, gastrointestinal symptoms, leukopenia, and elevated levels of serum hepatic enzymes. Patients with SFTS usually die from multiple organ failure, and the average fatality rate is 12%, although it has been reported to be as high as 30% in some areas [1–3]. SFTS was first reported in China, with additional cases subsequently confirmed in Japan, Korea, and, most recently, Vietnam [4]. Two cases with comparable symptoms caused by a similar virus, Heartland virus, were reported in the United States, and cases of infection with novel bandaviruses, including the Hunter Island group virus, Malsoor virus, and Guertu virus, were reported in Australia, India, and China, respectively [1, 5–10].

SFTS is mainly transmitted by ticks. Specifically, ticks of the family Ixodidae have been implicated as vectors of SFTSV. However, human-to-human transmission by contact with blood or body fluid from infected patients has also been reported in China and South Korea [2, 11–14]. Significantly, a novel case of SFTS infection was reported in South Korea without evidence of a tick bite [13]. Since the first report of SFTS infection in 2010, the number of cases has continuously increased every year in China, Japan, and South Korea. Patient surveillance in South Korea demonstrated 36 confirmed cases in 2013, which increased to 55 in 2014, 79 in 2015, and 165 in 2016 [15, 16]. A total of 158 SFTSV strains were isolated from the serum of these patients as described previously [17, 18]. Given the novelty of this virus and the limited information available, we aimed to acquire more molecular-level information on SFTSV toward the goal of developing a new diagnostic method for SFTS. To this end, we randomly selected 51 cases while ensuring that all provinces with a confirmed SFTS patient were included, and the isolates from these cases were sequenced.

The 51 clinical samples used in this study were collected as part of a laboratory surveillance system led by the Korea National Institute of Health (KNIH) during 2013–2016. In brief, the 5′- and 3′-terminal regions were sequenced by rapid amplification of cDNA ends technology. The genome sequences, including 41 tripartite (segments L, M, and S) and 10 bipartite (segments M and S) sequences, were generated using *de novo* assembly with DNASTAR SeqMan version 7.1 (Lasergene). The genome sequences obtained in this study were deposited in the GenBank/EMBL/DDBJ databases under the accession numbers KU507543–KU507577, KP663731–KP663745, and MF094728–MF094820, respectively. For gene characterization, we collected and manually edited 207 tripartite segmented genome sequences (163 Chinese, 43 Japanese, and one Korean) with available sampling dates from the GenBank database. Here, we focused on the protein-coding regions of SFTSV to investigate sequence variations and evolutionary dynamics.

The geographical distribution of the sequenced SFTSV samples is shown in Figure 1. In our dataset, isolates from Daegu represented the majority of the SFTSV genomes sequenced.

**Fig. 1:**
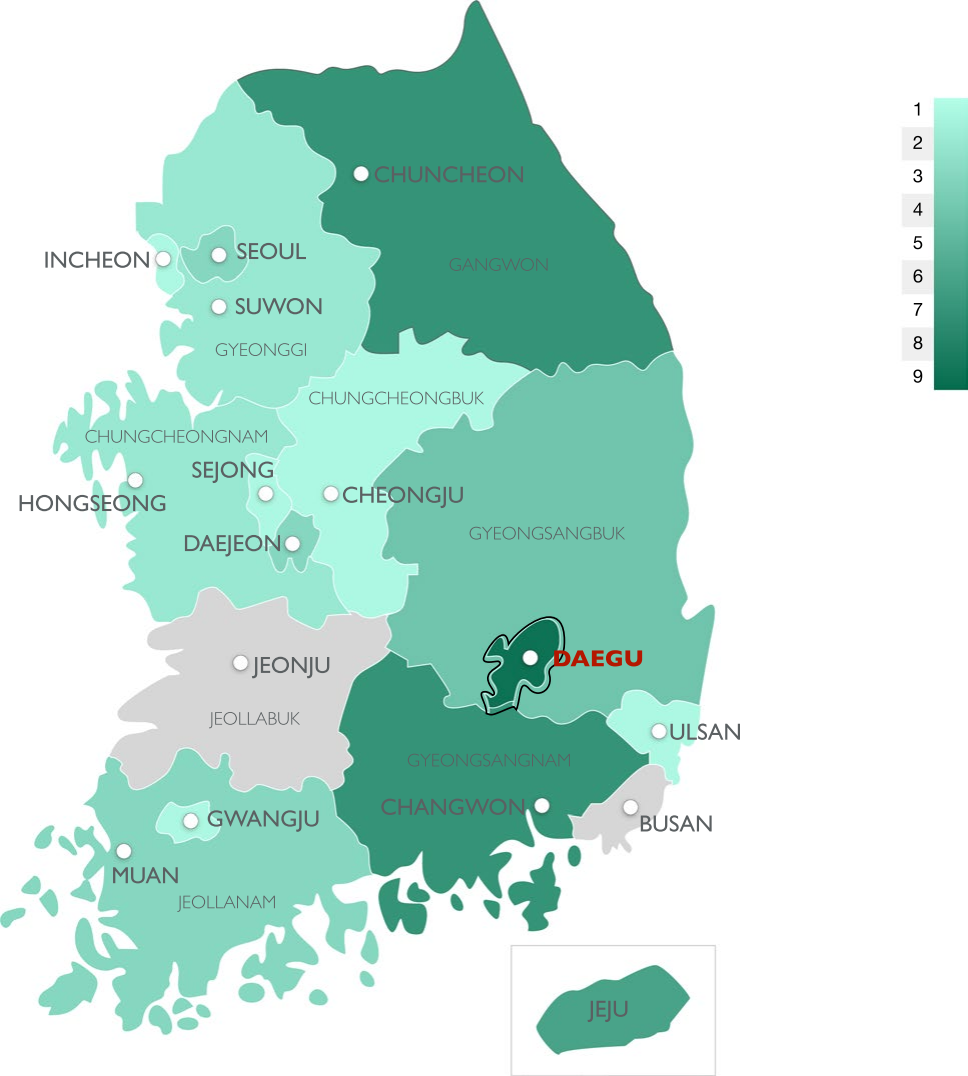
Geographic distribution of 51 selected SFTS cases in South Korea from 2013 to 2016 analyzed in this study. Of the regions with confirmed cases, Daegu was the main endemic region in this study. The shading of each region reflects the number of SFTS cases by area

Variation analysis was performed using 207 genome sequences collected from the National Center for Biotechnology Information GenBank database and the 51 genome sequences from the KNIH. The genome sequences were aligned against a reference genome sequence (strain HB29: accession no. NC_018139, NC_018138, and NC_018137 for the L, M, and S segment, respectively) using MUSCLE v3.8 [19]. At the nucleotide level, the total coding sequence length of the three segments was 6255, 3222, and 1620 nucleotides for the L, M, and S segment, respectively. This dataset revealed sequence variations by segment, including 1,254 variations for segment L, 803 for segment M, and 358 for segment S, 207, 154, and 58 of which were present exclusively in the Korean isolates, respectively.

At the amino acid level, the L, M, and S segments contain 2084, 1074, and 540 amino acid residues, respectively. In the Korean isolates, 82, 122, and 48 amino acids varied in the L, M, and S segments, respectively, 31, 37, and 16 of which were specific to the Korean sequences. In segment S, site 238 of the nonstructural protein coding region contained multiple variations: D (Asp) > E (Glu)/N (Asn)/G (Gly). In all of the Japanese sequences, this change was to E (Glu), whereas the Korean sequences presented three variations: one E (Glu) (strain 16KS28), two N (Asn) (strain 16KS31 and 16KS40), and one G (Gly) (strain 16KS26). A Japanese research group reported that substitution of the amino acid residue 962 (R > S) is crucial for the membrane fusion step of viral infection [20]. In our data, all of the KNIH strains except for strain 15KS7 (accession no. MF094809) had this replacement at residue 962. Another study found that the R > W 2 substitution at position 624 was associated with strong cell-fusion activity under acidic conditions, although none of the KNIH strains showed this variation [21].

To investigate the evolutionary dynamics of SFTSV, a maximum-clade-credibility tree was constructed from Bayesian phylogenetic analysis using the BEAST v1.8.4 package [22] and the FigTree v1.4 program [23], with general time-reversible, gamma-distributed substitution rate heterogeneity (G) and proportion of invariable sites (I) under both strict and uncorrelated relaxed molecular clocks. The trees for each of the three segments showed a similar topology (Fig. 2). A total of 248 sequences for segment L and 258 sequences for segments M and S were divided into two major geographical clades, designated as the Chinese clade and the Korean/Japanese clade (hereafter referred to as clade B, representing the virus commonly circulating in South Korea and Japan). The Chinese geographical clade was composed of five clades (A, C–F), and geographical clade B was the largest single clade.

**Fig. 2:**
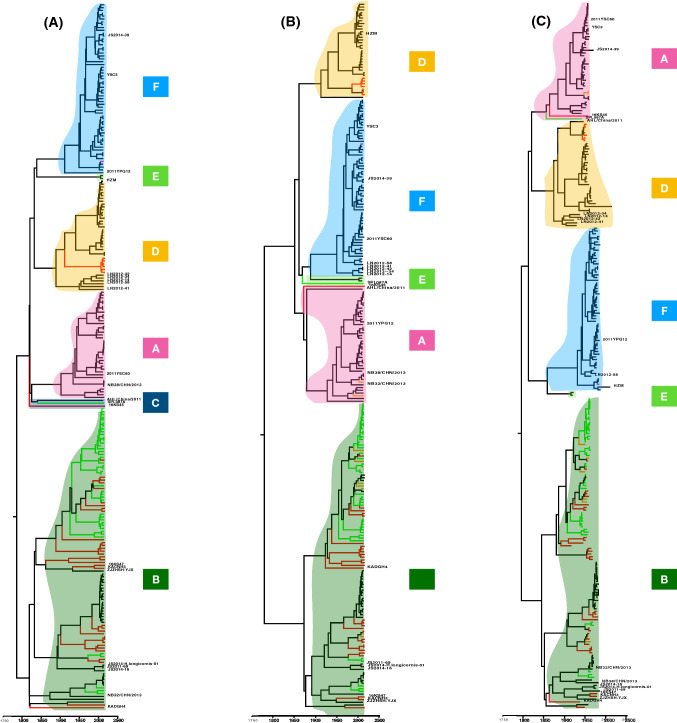
Maximum-clade-credibility phylogenetic trees of the three genome segments: (A) segment S, (B) segment M, and (C) segment L. Red, Korean complete genome sequences; orange, Korean incomplete genome sequences (M and S segments only); green, Japanese complete genome sequences; black, Chinese complete genome sequences; purple, Korean complete genome sequence from GenBank; blue, C1 clade in each of the three segments

Among all of the analyzed isolates in clade B, there were 30 Chinese, 42 Japanese, and 34 Korean strains for segment L; 29 Chinese, 42 Japanese, and 41 Korean strains for segment M; and 30 Chinese, 42 Japanese, and 41 Korean strains for segment S. Among the 41 tripartite KNIH genomes (segments L, M, and S), 34 were clustered in clade B. Of the bipartite KNIH sequences (segments M and S), seven of 10 isolates were also grouped in clade B. Six sequences of KNIH isolates – KASJH (2014), 16KS15 (2016), 16KS17 (2016), 16KS33 (2016), 16KS51 (2016), and 16KS52 (2016) – were grouped in the Chinese clade D, whereas one and two isolates of the remaining three bipartite-sequenced samples belonged to the Chinese clade D and A, respectively. One unique isolate from the KNIH (16KS45) was identified to have resulted from multiple inter-lineage reassortment. This isolate was grouped into different Chinese clades according to the segment analyzed: the segment L tree grouped 16KS45 in clade C, whereas the segment M and S trees grouped this isolate into clade A.

Although 98% of the Japanese isolates clustered in clade B, the isolate SPL087A grouped in Chinese clades; clade C for segment L, clade E for segment M, and clade A for segment S. For the Chinese isolates, 81.6% of the genome sequences clustered in the Chinese clades, whereas 30 isolates clustered in the Korean/Japanese clade B. Altogether, these results indicate that the majority of the Korean and Japanese SFTSV genomes cluster distinctly from the Chinese SFTSV genomes. Nevertheless, clade B may need to be separated into at least three subclades owing to the recent growth of this clade with a large number of Korean SFTSV sequences.

Genetic reassortment within the segmented RNA genome of SFTSV was observed in this study (Table 1). The Japanese isolate SPL087A emerged as a unique reassortant within the Japanese genomes and clustered in the Chinese clade C, E, and A for the L, M, and S segment, respectively. The Korean isolate 16KS45 was a unique reassortant among the Korean sequences, belonging to the Chinese clade C, A, and A for the L, M, and S segment, respectively. The Chinese strains NB32 and NB38 were reassigned from Chinese clades to the Korean/Japanese clade B. NB32 clustered in clade B, A, and B and NB38 clustered in clade A, A, and B for segment L, M, and S, respectively. Of the 15 strains that resulted from reassortment, eight had their L and S segments assigned to the same clade and the M segment was assigned to a different clade, which in accordance with the findings of Rezelj et al. [24]. The present analysis also identified a novel Korean reassortant of SFTSV that was not found in earlier studies [25, 26].

**Table 1:** Reassortants identified based on phylogenetic tree topology differences

Isolate	Country (city)	Collection date (year)	L	M	S
HZM	China (Huaiyangshan)	2010	D	D	F
2011YPQ12	China (Henan, Xinyang pingqiao)	2011	F	A	F
2011YSC60	China (Henan, Xinyang guangshan)	2011	A	F	A
YSC3	China (Henan, Xinyang)	2011	F	F	A
AHL/China/2011	China (Anhui)	2011	C	A	D
LN2012-14	China (Liaoning)	2012	D	F	D
LN2012-34	China (Liaoning)	2012	D	F	D
LN2012-41	China (Liaoning)	2012	D	F	D
LN2012-42	China (Liaoning)	2012	D	F	D
LN2012-58	China (Liaoning)	2012	D	F	F
NB32/CHN/2013	China (NA)	2013	B	A	B
NB38/CHN/2013	China (NA)	2013	A	A	B
JS2014-39	China (Jiangsu)	2014	F	F	A
SPL087A	Japan (NA)	2013	C	E	A
16KS45	South Korea (Gyeongsangnam)	2016	C	A	A

Bayesian phylogenetic analysis was performed to estimate the evolutionary rate and timescale for SFTSV. The evolutionary rate of all sequences of SFTSV was estimated to be 1.07E-4 (5.25 E-5–1.62E-4) for segment L, 2.08E-4 (1.11E-4–3.04E-4) for segment M, and 2.60E-4 (1.4588E-4–3.5961E-4) for segment S. The estimated time of the most recent common ancestor was 1736.66 (1566.24–1874.19) for segment L, 1758.65 (1600.62–1875.34) for segment M, and 1869.82 (1798.98–1929.03) for segment S, thereby indicating that SFTSV might have originated between 1736 and 1869. Although a different dataset was used in each study, our estimates of evolutionary rate were similar to those reported previously [27, 28]. However, Liu et al. [26] reported 3.25–4.2 times higher evolutionary rates than our estimates.

In summary, in this study, we determined 51 full-length genome sequences of Korean SFTSV isolates that were sampled from 2013 to 2016. This is the first phylogenetic and evolutionary analysis of a large number of Korean SFTSV genome sequences. Most of these KNIH sequences clustered in a major clade with Japanese sequences, whereas six complete KNIH genome sequences clustered in Chinese clades. One of the Korean isolates was identified as a novel reassortant and was assigned to a Chinese clade.

## References

[1] Yu XJ, Liang MF, Zhang SY, Liu Y, Li JD, Sun YL (2011). Fever with thrombocytopenia associated with a novel bunyavirus in China. N Engl J Med..

[2] Liu Y, Li Q, Hu W, Wu J, Wang Y, Mei L (2012). Person-to-person transmission of severe fever with thrombocytopenia syndrome virus. Vector Borne Zoonotic Dis.

[3] Zhang YZ, Zhou DJ, Qin XC, Tian JH, Xiong Y, Wang JB (2012). The ecology, genetic diversity, and phylogeny of Huaiyangshan virus in China. J Virol.

[4] Tran XC, Yun Y, An LV, Kim SH, Thao NTP, Man PKC (2019). Endemic severe fever with thrombocytopenia syndrome, Vietnam. Emerg Infect Dis.

[5] Takahashi T, Maeda K, Suzuki T, Ishido A, Shigeoka T, Tominaga T (2014). The first identification and retrospective study of severe fever with thrombocytopenia syndrome in Japan. J Infect Dis.

[6] Kim K-H, Yi J, Kim G, Choi SJ, Jun KI, Kim N-H (2013). Severe fever with thrombocytopenia syndrome, South Korea, 2012. Emerg Infect Dis.

[7] McMullan LK, Folk SM, Kelly AJ, MacNeil A, Goldsmith CS, Metcalfe MG (2012). A new phlebovirus associated with severe febrile illness in Missouri. N Engl J Med.

[8] Wang J, Selleck P, Yu M, Ha W, Rootes C, Gales R (2014). Novel phlebovirus with zoonotic potential isolated from ticks, Australia. Emerg Infect Dis.

[9] Mourya DT, Yadav PD, Basu A, Shete A, Patil DY, Zawar D (2014). Malsoor virus, a novel bat phlebovirus, is closely related to severe fever with thrombocytopenia syndrome virus and Heartland virus. J Virol.

[10] Shen S, Duan X, Wang B, Zhu L, Zhang Y, Zhang J (2018). A novel tick-borne phlebovirus, closely related to severe fever with thrombocytopenia syndrome virus and Heartland virus, is a potential pathogen. Emerg Microbes Infect.

[11] Chen H, Hu K, Zou J, Xiao J (2013). A cluster of cases of human-to-human transmission caused by severe fever with thrombocytopenia syndrome bunyavirus. Int J Infect Dis.

[12] Jiang XL, Zhang S, Jiang M, Bi ZQ, Liang MF, Ding SJ (2015). A cluster of person-to-person transmission cases caused by SFTS virus in Penglai, China. Clin Microbiol Infect.

[13] Kim WY, Choi W, Park SW, Wang EB, Lee WJ, Jee Y (2015). Nosocomial transmission of severe fever with thrombocytopenia syndrome in Korea. Clin Infect Dis.

[14] Tang X, Wu W, Wang H, Du Y, Liu L, Kang K (2013). Human-to-human transmission of severe fever with thrombocytopenia syndrome bunyavirus through contact with infectious blood. J Infect Dis.

[15] KCDC Releases 2016 Infectious disease surveillance yearbook (in Korean). http://www.cdc.go.kr/npt/biz/npp/portal/nppPblctDtaView.do?pblctDtaSeAt=1&pblctDtaSn=22. Accessed 1 June 2017

[16] Choi SJ, Park S-W, Bae I-G, Kim S-H, Ryu SY, Kim HA (2016). Severe fever with thrombocytopenia syndrome in South Korea, 2013–2015. PLoS Negl Trop Dis.

[17] Park SW, Han MG, Yun SM, Park C, Lee WJ, Ryou J (2014). Severe fever with thrombocytopenia syndrome virus, South Korea, 2013. Emerg Infect Dis.

[18] Park S-W, Ryou J, Choi W-Y, Han M-G, Lee W-J (2016). Epidemiological and clinical features of severe fever with thrombocytopenia syndrome during an outbreak in South Korea, 2013–2015. Am J Trop Med Hyg.

[19] Edgar RC (2004). MUSCLE: multiple sequence alignment with high accuracy and high throughput. Nucleic Acids Res.

[20] Tani H, Kawachi K, Kimura M, Taniguchi S, Shimojima M, Fukushi S (2019). Identification of the amino acid residue important for fusion of severe fever with thrombocytopenia syndrome virus glycoprotein. Virology.

[21] Tsuda Y, Igarashi M, Ito R, Nishio S, Shimizu K, Yoshimatsu K (2017). The amino acid at position 624 in the glycoprotein of SFTSV (severe fever with thrombocytopenia virus) plays a critical role in low-pH-dependent cell fusion activity. Biomed Res.

[22] Drummond AJ, Suchard MA, Xie D, Rambaut A (2012). Bayesian phylogenetics with BEAUti and the BEAST 1.7. Mol Biol Evol.

[23] Rambaut A (2014) Figtree, a graphical viewer of phylogenetic trees. http://tree.bio.ed.ac.uk/software/figtree

[24] Rezelj VV, Mottram TJ, Hughes J, Elliott RM, Kohl A, Brennan B (2019). M segment-based minigenomes and virus-like particle assays as an approach to assess the potential of tick-borne *Phlebovirus* genome reassortment. J Virol.

[25] Shi J, Hu S, Liu X, Yang J, Liu D, Wu L (2017). Migration, recombination, and reassortment are involved in the evolution of severe fever with thrombocytopenia syndrome bunyavirus. Infect Genet Evol.

[26] Lv Q, Zhang H, Tian L, Zhang R, Zhang Z, Li J (2017). Novel sub-lineages, recombinants and reassortants of severe fever with thrombocytopenia syndrome virus. Ticks Tick Borne Dis.

[27] Liu JW, Zhao L, Luo LM, Liu MM, Sun Y, Su X (2016). Molecular evolution and spatial transmission of severe fever with thrombocytopenia syndrome virus based on complete genome sequences. PLoS One.

[28] Fu Y, Li S, Zhang Z, Man S, Li X, Zhang W (2016). Phylogeographic analysis of severe fever with thrombocytopenia syndrome virus from Zhoushan Islands, China: implication for transmission across the ocean. Sci Rep.

