# Correction to: Genetic diversity and evolutionary history of Korean isolates of severe fever with thrombocytopenia syndrome virus from 2013–2016

**DOI:** 10.1007/s00705-020-04775-4

**Published:** 2020-09-11

**Authors:** Mi-ran Yun, Jungsang Ryou, Wooyoung Choi, Joo-Yeon Lee, Sun-Whan Park, Dae-Won Kim

**Affiliations:** 1 Pathogen Resource TF, Center for Infectious Diseases Research, Korea National Institute of Health, Korea Centers for Disease Control and Prevention, 200 Osongsaengmyeong2-ro, Heungdeok-gu, Cheogju-si, Chungbuk 28160 Republic of Korea; 2 Division of Emerging Infectious Disease and Vector Research, Center for Infectious Diseases Research, Korea National Institute of Health, Korea Centers for Disease Control and Prevention, 187 Osongsaengmyeong2-ro, Heungdeok-gu, Cheogju-si, Chungbuk 28160 Republic of Korea; 3 Division of Viral Diseases, Center for Laboratory control of Infectious Disease, Korea Centers for Disease Control and Prevention, 187 Osongsaengmyeong2-ro, Heungdeok-gu, Cheogju-si, Chungbuk 28160 Republic of Korea; 4 Jeju National Quarantine Station, Korea Centers for Disease Control and Prevention, 356 Central Goverment office-Jeju, 59 Cheongsa-ro, Jeju-si, 63219 Republic of Korea

## Correction to: Archives of Virology 10.1007/s00705-020-04733-0

Authors would like to correct the 4th author name from “Ju-Yeon Lee” to the correct version “Joo-Yeon Lee”.

The original article has been corrected.

